# Cultural adaptation of INDIGO mental health stigma reduction interventions using an ecological validity model in north India

**DOI:** 10.3389/fpsyt.2024.1337662

**Published:** 2024-01-31

**Authors:** Mercian Daniel, Sudha Kallakuri, Petra C. Gronholm, Syed Shabab Wahid, Brandon Kohrt, Graham Thornicroft, Pallab K. Maulik

**Affiliations:** ^1^ Research Department, George Institute for Global Health, New Delhi, India; ^2^ Centre for Global Mental Health and Centre for Implementation Science, Health Service and Population Research Department, Institute of Psychiatry, Psychology & Neuroscience, King’s College London, London, United Kingdom; ^3^ Department of Global Health, School of Health, Georgetown University, Washington, DC, United States; ^4^ Center for Global Mental Health Equity, The George Washington University School of Medicine and Health Sciences, Washington, DC, United States; ^5^ Faculty of Medicine, University of New South Wales, Sydney, NSW, Australia; ^6^ Department of Brain Sciences, Imperial College London, London, United Kingdom; ^7^ Department of Public Health, Prasanna School of Public Health, Manipal Academy of Higher Education, Manipal, India

**Keywords:** indigo, cultural adaptation, mental health, stigma reduction, discrimination, India

## Abstract

**Background:**

The International Study of Discrimination and Stigma Outcomes (INDIGO) Partnership is a multi-country international research program in seven sites across five low- and middle-income countries (LMICs) in Africa and Asia to develop, contextually adapt mental health stigma reduction interventions and pilot these among a variety of target populations. The aim of this paper is to report on the process of culturally adapting these interventions in India using an established framework.

**Methods:**

As part of this larger program, we have contextualized and implemented these interventions from March 2022 to August 2023 in a site in north India. The Ecological Validity Model (EVM) was used to guide the adaptation and contextualization process comprising eight dimensions.

**Findings:**

Six dimensions of the Ecological Validity Model were adapted, namely language, persons, metaphors, content, methods, and context; and two dimensions, namely concepts and goals, were retained.

**Conclusion:**

Stigma reduction strategies with varied target groups, based on culturally appropriate adaptations, are more likely to be acceptable to the stakeholders involved in the intervention, and to be effective in terms of the program impact.

## 1 Introduction

Stigma and discrimination towards people with mental health conditions are universally reported across the world (1). People with mental health conditions experience stigma, prejudice, and discrimination from a variety of different sources and settings. Such stigma can come, for example, from the public and local communities where people with mental health conditions live (2, 3), from primary care workers and the healthcare system (4, 5), or from specialist mental health professionals (6). Stigma has wide-ranging consequences on people with mental health conditions, who have described stigma as worse than the mental health condition itself (1, 7). Stigma from different sources can have a range of deleterious impacts on people with mental health conditions, who can be excluded from social participation, with negative impacts on wellbeing, reduced employment opportunities, greater risk of poverty, difficulties in maintaining personal relationships (8), and limited access to health care because of barriers to help seeking (9, 10).

There is limited research conducted in low-and middle-income countries (LMICs) that evaluates the effectiveness of interventions to reduce mental health stigma and discrimination among groups that have the potential to stigmatize (11–14). There are studies in some of these countries that have shown an increase in the uptake of mental health services following an anti-stigma and awareness campaign (15, 16). There is also emerging evidence that basic mental health services can be provided by trained non-specialists (e.g., primary care providers and community health workers, peers, and other lay persons) in LMICs (17, 18). However, there is an absence of studies on anti-stigma interventions that target mental health professionals in LMICs (12), with relevant studies mostly taking place in high-income countries (19, 20). Recent studies and systematic reviews examining interventions to reduce mental health stigma and discrimination have shown that there is an increasing number of effective interventions being reported from LMICs (11–14). There is more evidence on interventions to change mental health knowledge, but fewer that addresses the critical areas of attitudes and behavior (11, 12). Extant evidence on stigma interventions has shown the effectiveness of social contact-based interventions in reducing mental health stigma, with regard to changes in discriminatory behaviors across different target groups (1). However, this evidence is less robust from LMICs (4, 11, 21) where the cultural contexts and stigmatizing groups can be very diverse.

The ways in which stigma and discrimination are expressed by different groups at the community, primary healthcare, and mental health system levels, as well as how they are experienced by people with mental health conditions, varies a great deal across countries and cultures (22–25). Dominant local beliefs about mental health conditions co-exist along with western biomedical models in LMICs with a pluralistic health system (26, 27). Labels, stereotypes, and causal attributions attached to mental health conditions vary considerably across different cultures (22, 28), and may mediate the extent to which people with mental health conditions are stigmatized and seek treatment (29). A study conducted in North India showed that the most commonly used labels for people with severe mental illness were *“Aalsi”* (lazy), *“sustt”* (lethargic), *“paagal”* (mad), and *“darpok”* (coward). About one third of the participants with these illnesses reported experiencing these labels were significantly associated with discontinuing treatment or a desire to do so (29). Despite such cultural variations, promising research shows that evidence-based psychological interventions are effective for the treatment of mental health conditions among diverse populations (30, 31). A recent review that examined incorporating cultural elements into anti-stigma programs in LMICs found that only a fifth of the studies considered cultural values, meanings, and practices in their interventions with healthcare professionals, community members and people with lived experiences (32).

In this context, the International Study of Discrimination and Stigma Outcomes (INDIGO) Partnership is a multi-country international research program in seven sites across five LMICs in Africa and Asia to develop, test and contextually adapt mental health stigma reduction interventions among a variety of target populations (33). The three interventions that are being piloted target community members and community health workers (CHWs) (Indigo-Local) (34), primary care providers (PCPs) (Indigo-Primary) (35), and mental health professionals (Indigo-READ) (36). As part of this larger program, we have contextualized and implemented these interventions in a site in north India. The aim of this paper is to report the process of culturally adapting these interventions using an established framework.

## 2 Methods

### 2.1 Study implementation and design

Implementation of the Indigo-Primary intervention among PCPs was the first of the three interventions to be implemented. This included two site staff (MD and SK), i.e. researchers involved in mental health projects, being trained in the RESHAPE (REducing Stigma among HealthcAre ProvidErs) manual (35, 37), a social-contact based stigma reduction intervention, in which five mental health service users (MHSUs) and two caregivers were trained in the photovoice technique over a period of two-and-a-half months. This is a participatory photography narrative technique to visually develop and narrate recovery stories and testimonials (38). The intervention included training primary health care providers on mental health where social contact of photovoice trained MHSUs was integrated. Two health workers were trained in the WHO mhGAP-Intervention Guide (depression and suicide modules) (39) where the two MHSUs and their caregivers narrated their recovery stories using the photovoice technique, along with a model aspirational figure (primary healthcare facility doctor) who had experience of managing people with common mental disorders. The aspirational figure shared her reflections and learnings from providing treatment for common mental disorders in a primary health care setting.

The Indigo-Local intervention comprised of a community-based, multi-component, public awareness-raising activities designed to reduce stigma and discrimination and to increase referrals of people with mental health conditions. Implementation of the intervention among community members and CHWs involved organizing two stakeholder meetings where a total of more than 50 participants were present, comprising local administrative officials of different political parties, schoolteachers, CHWs, health workers, and community members. Eleven CHWs and two MHSUs participated in the intervention trainings. A media campaign for this study was rolled out in the community using printed materials and lived experience videos developed as a part of the SMART mental health study, where a large anti-stigma campaign was implemented in a similar setting (40). This campaign as a part of INDIGO Local, was implemented for three months and covered 1185 community members with majority (70%) being women.

The Indigo-READ intervention assesses the feasibility, potential effectiveness and costs of responding to experienced and anticipated discrimination training for health professionals working in mental health care. This intervention included in-person training of six mental health professionals of the district mental health program comprising of two psychiatric nurses, one psychologist, one psychiatrist, a data entry personnel, and an intern. In addition to a service user who presented her personal testimony as an expert by experience, a short video of a person with lived experience was screened in the training.

All three interventions employed an uncontrolled design with evaluations pre-and post-intervention, and a later follow up point at three, six- or 12-months’ time. These are small scale feasibility studies that test a particular proof-of-principle largely employing mixed methods approaches (41). **Box 1** describes the different program details and key components of each of the Indigo interventions. **Table 1** provides further details on the methodological aspects of each intervention in the North Indian site. All the three interventions have been completed and there is a plan to bring out separate site-specific publication following the main and combined publication of each of these interventions by the work package leads.

**Table 1:** Study design, sample size, follow-up evaluations and methods for Indigo interventions in North India.

Interventions (Work Package)	Study design	Sample characteristics and size	Follow-up evaluations and methods
Reducing stigma and improving access to care for people with mental health conditions in the community (Indigo-Local)	Feasibility pilot	11 Accredited Social Health Activists (ASHAs); 3 service users.	Quantitative- pre, post, 6 and 12 months Qualitative- 6 months
Contact-based intervention to reduce mental health related stigma in primary healthcare setting (Indigo-Primary)	Uncontrolled before-after proof-of-principle	5 service user, 3 primary care provider (PCP)	Quantitative- pre, post, 3 and 6 months Qualitative- 6 months
Mental health professionals responding to experienced and anticipated mental health related discrimination (Indigo-READ)	Uncontrolled pre-post feasibility	5 mental health professionals	Quantitative- pre, post and 3 months Qualitative- post and 3 months

Box 1 **Program details and key components of Indigo interventions.**

**Indigo-Local**

- **Aim**- to develop, implement, and evaluate a community-based, multi-component, public awareness-raising intervention designed to reduce stigma and discrimination and to increase referrals of people with mental health conditions for assessment and treatment.
- **Key components**- stakeholder group workshop; a stepped training programme of CHWs and MHSUs with repeated supervision and booster sessions; awareness raising activities in the community; and a media campaign.
- Social contact and service user involvement are instrumental to all components.
- **Evaluation**- mixed-methods pre-post design; quantitative assessment of stigma outcomes measuring knowledge, attitudes, and behaviour; quantitative evaluation of mental health service utilization rates; qualitative assessment of effectiveness and impact of the intervention; process evaluation; implementation evaluation; and evaluation of implementation costs.
- **Sample size**- 11-86 CHWS, 3-5 MHSUs, and 5-20 stakeholders. Participants were sampled purposively and based on feasibility and availability of local resources and size of the site.

**Indigo-Primary**

- **Aim**- to adapt and evaluate cross-cultural feasibility and acceptability of a social contact-based primary healthcare intervention.
- **Key components**- collaboration MHSUs with lived experience of mental health conditions, their family members, and aspirational figures (PCPs who have demonstrated high motivation to integrate mental health services). MHSUs and their family members are trained in a participatory technique, PhotoVoice, to visually depict and narrate recovery stories. Aspirational figures conduct myth busting exercises and share their experiences treating MHSUs.
- **Evaluation**- uncontrolled before-after study design; outcomes among PCPs will include stigma knowledge, explicit and implicit attitudes, and mental healthcare competencies; qualitative interviews with MHSUs, family members, and aspirational figures, PhotoVoice trainers, mental health specialists co-leading the primary care trainings, and PCPs receiving mental health training; generate evidence regarding feasibility, acceptability, recruitment, retention, fidelity, safety, and usefulness of the intervention.
- **Sample size**- 6-20 MHSUs, 1-8 aspirational figure, and 2-36 PCPs. The sample size were determined by the number of participants available and feasibility to include them in the study.

**Indigo-READ**

- **Aim**- assess feasibility, potential effectiveness, and costs of Responding to Experienced and Anticipated Discrimination training for health professionals working in mental health care.
- **Key components**- training draws upon evidence bases for stigma reduction, health advocacy and medical education and is tailored to sites through situational analyses; content, delivery methods and intensity were agreed upon through a consensus exercise with site research teams; delivered to health professionals working in mental health care.
- **Evaluation**- uncontrolled pre-post mixed methods feasibility study; baseline data collection; outcome measures at post-training and 3 months post-baseline, followed by qualitative data collection; fidelity rated during intervention delivery; data on training costs; qualitative data to identify feedback about training methods and content, including the implementability of the knowledge and skills learned; pooled and site-specific training costs per trainee and per session.
- **Sample size**- 4-30 mental health professionals. As this is a feasibility study without any control group the sample size is not designed to determine effectiveness.

### 2.2 Setting

The interventions were conducted in and around two Urban Primary Health Centres (UPHCs) of Atmadpur and Mewla Maharajpur, covering the areas of Rajeev nagar, Harkesh nagar, Santosh nagar, Dheeraj nagar part 1, Dheeraj nagar part 2, under the former UPHC and Mewla Maharajpur, Ghandhi colony, Fatehpur Chandela, under the latter UPHC. These UPHCs fall under the district of Faridabad, Haryana state where the intervention with mental health professionals took place at the district hospital. The UPHCs and district hospital were chosen as they were near the central office and field offices of the implementing site of Delhi, and we had a well-established relationship with the state and district health departments. Study participants across all interventions were selected using a qualitative technique akin to convenience sampling as they were easily accessible, and such a technique is generally applied for pilot testing (41).

The activities across all the three anti-stigma interventions started in July 2022 with the integrated training of PCPs beginning first. However, the preparation for this intervention started much before in March 2022 with the training of service users on the photovoice technique. All intervention activities have been completed with a final quantitative evaluation of CHWs done in late August 2023.

### 2.3 Cultural adaptation framework

Each of the Indigo interventions went through a process of adaption. The adaptations were specifically made keeping in mind the local cultural context in which the interventions were implemented. The Ecological Validity Model (42) was used to document the adaptation and contextualization process. This documentation took place either during the preparatory phase of planning the interventions or during and after certain intervention activities were completed. The EVM outlines eight contextualization dimensions, with operational definitions provided in **Table 2**.

**Table 2:** Operational definitions of EVM dimensions.

Dimensions	Definitions
1. Concepts	Concepts refer to how the intervention material is thought of and communicated to the facilitators, intervention participants, community members, and other stakeholders. The programs’ and the facilitators’ credibility may be reduced if the communication of concepts and the concepts themselves do not match the local culture.
2. Methods	Methods are the procedures followed to achieve treatment goals. These methods and procedures should be congruent with the participants’ culture and use of language.
3. Goals	Goals are the agreement between participants and facilitator in what participants would like to achieve during the course of the intervention. These goals must be realistic and fit with the participants’ values and personal motivations.
4. Context	Context refers to the participants’ economic, social, political and cultural environment. This should look beyond just the participant as an individual and focus on outside factors, such as socialization, discrimination, and family history, that could influence the intervention.
5. Content	The knowledge, values, customs, and traditions shared by the participants should be integrated into all elements of the intervention. This can be seen as a starting point for culturally adapting the recruitment process, assessments, and the treatment itself.
6. Metaphors	Culturally appropriate symbols or concepts should be embedded within the intervention that support participants in absorbing the intervention’s core mechanisms of action. Metaphors used may be pictorial, idioms, commonly used phrases or item and symbols.
7. Persons	A culturally appropriate intervention must consider the role of ethnicity, race, gender, class and other relevant social constructs in the relationship between the participants and facilitators. This relationship should respect expectations and limitations that are reflective of the local culture.
8. Language	Language is inherently attached to culture and is related to the expression of emotional experiences. The intervention should be in the language most comfortable and accessible to the participants and should also use appropriate terminology based on the education levels of the facilitators and participants.

Across each of these dimensions six domains of contextualization were recorded: 1) original content changed, 2) pages/location in the intervention manual or materials, 3) description of contextualization, 4) rationale of change and what it would accomplish, 5) evidence for change, and 6) the source of this evidence. Characteristics of team members who conducted the contextualization procedure were also recorded. This included indicating relevant educational qualifications and work experience of the team members, and any other relevant demographic information. Additionally, it was critically important to mention whether team members spoke the local language and had experience working in settings where the intervention was rolled out.

### 2.4 Characteristics of team conducting cultural adaptation and contextualization

A total of three staff members were involved in the cultural adaptation process with diverse educational qualifications, experience, genders, and capability of speaking in the local language of study participants. The educational qualifications of the team members varied from Masters to Post-doctoral level in social work or public health. Experience of working in the field of mental health also varied among the team members between 5-20 years. All the members involved in the cultural adaptation process had varying capabilities of reading, writing, and speaking the local language of study participants. Two of the team members (SK and SC) could read, write, and speak the local language and were involved in the translation of training materials from English to Hindi and conducting the training sessions.^1^ MD adapted the training materials as per the need and context of the interventions that were provided in English by the work package leads. These were then handed over to SC who was primarily involved in translating the materials to Hindi. This was then cross checked and finalized by SK. If any disagreements on the translation arose, then it was consensually decided by both SC and SK. One team member (MD) who could speak the local language was involved in conducting the training sessions.^1^ All team members had the experience of directly engaging with MHSUs from an ongoing project (40, 43).

## 3 Results

### 3.1 Cultural adaptation and contextualization

The processes of culturally adapting the interventions are documented and presented in a matrix under each contextual dimension and across the different domains (**Table 3**). All materials used in the training of MHSUs and CHWs that were presented in the form of power point slides were originally developed in English and then translated into Hindi (the local vernacular language spoken in the area). These changes have been indicated in the pages of the original training manuals and the adapted manuals or power point slides. Training materials for mental health professionals (MHPs) were however retained in the original English language and used as such, though much of the discussion around the content of the training were done in the local Hindi language.

**Table 3:** Cultural adaptation of Indigo study interventions using an ecological validity model matrix.

Adaptation Dimension	Original Content	Pages/Location	Contextualization Strategy	Rationale	Evidence	Source
Language	• Terms used for mental health and mental illness, severe mental disorders, depression and anxiety, stigma, discrimination and CHWs	• Sections in original training materials • Adapted training manual • Adapted PPT training slides	Terms adapted: • Mental health= *“mansik swasth”* • Mental illness= *“mansik rog”* • Severe mental disorders= *“gambhir mansik rog”* • Depression= *“udasi”* • Anxiety= *“ghabrahat”* • Stigma=*“kalank”* • Discrimination= *“bhedbhaw”* • CHWs= ASHAs	• These terms are locally relatable to study participants and conveys the intended meaning in the north Indian context	• Situational analysis in north Indian site • Cultural adaptation work of Indigo study • Literature review	• Kaur et al., 2023 (44) • Internal documents of Indigo study • Daniel et al., 2023 (45)
People	• Selection of CHWs, MHSUs, PCP, and MHPs	• Sections in original training materials • Adapted training manual	• Recruited ASHAs who work in areas serviced by two UPHCs • Doctors from these UPHCs • MHSUs with depression, anxiety and suicide risk who were women and from local or neighboring areas • MHPs (psychiatrist, psychologist, community, and psychiatric nurse) who work in DMHP and are posted at district hospital	• The site team had experience working with ASHAs • Some MHSUs was part of an existing project • There is a dearth of MHPs in district • Team of MHPs of DMHP, some of whom we have been working with in other mental health projects were available and accessible	• Situational analysis in north Indian site • Literature review	• Kaur et al., 2023 (44) • Internal documents of Indigo study. • Daniel et al., 2021 (40) • Daniel et al., 2023 (45)
Metaphors	• Introduction to mental health and mental illness	• Sections in original training materials • Adapted training manual • Adapted PPT training slides	• An in-house developed culturally adapted IEC material in lay local language with illustrations to orient CHWs and MHSUs was used • Similar materials were used for media campaign	• There were readily available materials adapted to the local context with simple and culturally relevant illustrations	• Literature review	• Kaur et al., 2021 (12) • Daniel et al., 2021 (40) • Daniel et al., 2023 (45)
Content	• Human Rights and mental illness • Service user rights and movement • Common myths surrounding mental illness • Aspirational figure presentation • Case studies	• Sections in original training materials • Adapted training manual • Adapted PPT training slides	• Case studies were adapted with changes in names and places, and existing context of the district health system • An example of successfully treating a person with depression was included • Salient points of Mental Health Care Act, 2017, and Mental health insurance policy relevant to MHSUs in the Indian context were incorporated • Adapted to include four common myths around mental illness, e.g., mental illness is caused by black magic or by evil spirits and were used during the trainings	• These adaptations are found to be culturally appropriate to training participants which they can relate to in their present context and discuss these more meaningfully • Adaptations of mental health policies are appropriate to the Indian context and is critical in discussions around MHSUs rights and policies • Positive stories would inspire other participants as they can relate with a real-life example of a MHSU being treated	• Situational analysis in north Indian site • Cultural adaptation work of Indigo study • Literature review	Kaur et al., 2023 (44) and internal documents of Indigo study; Daniel et al., 2023 (45)
Methods	• Duration and no. of sessions for study participants’ training program	• Sections in original training materials • Adapted training manual • Adapted PPT training slides	• Training timings were reduced, and the no. of sessions were also truncated to accommodate schedule of participants • For e.g., all 5 modules were covered for the MHPs’ training, but it was reduced from 6:30 to 3:15 hours • Used a short video of a young man with alcohol use and depressive disorders narrating his recovery story from an earlier study in a similar geographical location	• CHWs had additional responsibilities of implementing national programs on mother and child health • MHSUs were women with childcare and household responsibilities • PCPs have a busy schedule with responsibilities of implementing the national health programs • In addition to having MHSU with anxiety and depression who was a married woman with children, having a young single man with substance use disorder and depression will add to the diversity of testimonies and methods used in training program	• Situational analysis in north Indian site • Cultural adaptation work of Indigo study • Literature review	• Kaur et al., 2023 (44) • Internal documents of Indigo study • Daniel et al., 2021 (40) • Daniel et al., 2023 (45)
Context	• Recruitment of study participants	• Original training manual • Adapted training manual	• Recruited ASHAs being mindful of their job responsibilities • Recruited MHSUs keeping in mind their cultural context • Considering busy schedule of MHPs, duration of training was limited to one half of a day when they were free from their clinical duties	• ASHAs and MHSUs who consented to devote time for trainings and subsequent intervention activities were included • MHSU men who had initially joined training had to drop out because of employment commitments • Training timings had to be adjusted to accommodate schedule of participants	• Situational analysis in north Indian site • Cultural adaptation work of Indigo study • Literature review	• Kaur et al., 2023 (44) • Internal documents of Indigo study • Daniel et al., 2021 (40) • Daniel et al., 2023 (45).

### 3.2 Language

The adaptation of *language* in the training and intervention materials included terms used for mental health and mental illness, severe mental disorders, depression and anxiety, stigma, and discrimination. The translated Hindi terms used for these are further described in **Table 3**. The contextualization process considered previous research that was conducted in similar geographical locations and cultural context (40, 45) to where the Indigo interventions were implemented. Additionally, MHSUs were asked to elucidate derogatory terms used for people with mental health conditions that either they have been referred to or have heard somewhere. These terms were *“besahoor”* (creep), *“chatka huaa”* (cracked), *“paagal”* (mad), *“nasamaj”* (foolish), *“budhiheen”* (brainless), mental, *“ganwar”* (uneducated), *“bewakoof”* (idiot). It was necessary to bring out culturally dominant and derogatory stereotypes that MHSUs experienced to provide alternative destigmatizing terms to challenge these. These terms were adapted to the local context as they are more relatable to ASHAs and MHSUs and convey the desired meaning in the local context.

### 3.3 Persons

Participants who were recruited for the interventions were CHWs known as Accredited Social Health Activists (ASHAs) in India. As this was a pilot intervention, only few ASHAs who worked in select areas serviced by two UPHCs were included. ASHAs are an all women cadre of primary health workers and act as a link between the community and the health system (40, 46). The project team had experience working with ASHAs for a considerable period in mental health projects that also utilized an anti-stigma campaign (16, 40). The research team had experience working with MHSUs (40, 43). MHSUs with depression, anxiety, and suicide risk who were receiving treatment from either a public or private facility and those from an ongoing project were included in the interventions. Doctors from the UPHCs and mental health professionals who are a part of the District Mental Health Program (DMHP) at the district hospital were included in the trainings.

### 3.4 Metaphors

Culturally adapted materials in a lay local language that were used in an ongoing mental health study were used for the training of CHWs and MHSUs to orient them on mental health, types of mental disorders, causes of mental disorders, identifying mental disorders, treatment of common mental disorders (like depression, anxiety and suicide risk), ways to manage people with CMDs and ways to overcome stigma (45, 47). As part of a media campaign for the intervention with community members (Indigo Local), we used existing culturally adapted materials like a flipbook and a video of a person with lived experience. The flipbook included simple illustrations of what CMDs are and what can be done to overcome and live with CMDs. In the training with MHPs, we used materials in English as training participants and trainers spoke both English and Hindi. However, the discussions during the training were done in the local language, where for example local metaphors were used to explain the idea of empathy in a culturally relevant way by one of the participants.

### 3.5 Content

The intervention and training content used several case studies to substantiate and prompt discussions on aspects related to mental health and mental health stigma. The case examples were adapted with changes in names, places, and health system context to assist study participants relate to these in a more meaningful way. For instance, a real-life example of successfully treating a person with a mental health condition was narrated by a primary care provider. Based on a situational analyses study and earlier formative research, common myths of mental illness prevalent in the study area were adapted (44, 45) and included in the intervention trainings. Policies relevant to MHSUs in the Indian context were included and discussed in the trainings, which is critical in any discussion around MHSUs representation and rights.

### 3.6 Methods

There was no major change in the methods of imparting trainings for study participants as per the intervention protocol templates, which included didactic lectures, group exercises, case studies, and individual presentations. However, the duration of the trainings was reduced with the number of sessions shortened. The timings of the trainings were also scheduled in a way that accommodated the caring and household responsibilities of MHSUs, community outreach work of ASHAs, and clinical commitments of PCPs and MHPs. Trainings with participants who were extremely busy with their day-to-day work were conducted at the end of the program. In addition to using lived experience testimonies of MHSUs, we included a short video of a lived experience testimony to add to the diversity of the methods used in the intervention trainings.

### 3.7 Context

The research team conducting the interventions with different groups of study participants were mindful of their background, which included both their personal and professional context. Only those participants who gave their consent and agreed to take part and continue in the trainings were recruited. MHSUs who were men, for instance, could not continue in the trainings and intervention as they had to take care of their job commitments. The context of the study participants across all categories, i.e., mental health service users, primary care providers, community health workers, and mental health professionals, to a large extent had a bearing on the training methods employed in the intervention (**Table 3**).

### 3.8 Concepts and goals

While six dimensions of the intervention were adapted to the local context, the dimensions of ‘concepts’ and ‘goals’ were not changed and retained as they were. It was not necessary to change the different concepts that went into the Indigo interventions. The idea to engage with key community stakeholders prior to intervention activities to gain buy-in has been integral to the work that we do. During the stakeholders meetings we took feedback from community members on the strategies for the Indigo-Local media campaign. The social contact element was incorporated into all the interventions (33). The social-contact based concept of engagement in Indigo-Primary where PCPs interacted with SUs and aspirational figures in reducing potential survival, social and professional threats (37) also applied in our setting. Additionally, the concept of reflective practice, intergroup contact theory, and mechanisms that sustain social distance remained the same in the Indigo-READ intervention that involved mental health professionals (48, 49). There was no change in the goals of stigma reduction across all study groups, especially in terms of integrating a social contact element in the interventions.

## 4 Discussion

This study used a cultural framework to contextually adapt mental health stigma reduction interventions among community members and community health workers, PCPs, and mental health professionals in a north Indian site as part of the larger Indigo Partnership study. Guided by the EVM framework, six dimensions (language, persons, metaphors, content, methods, context) were adapted either before, during or after the interventions were implemented. However, two dimensions, (concepts and goals) were retained and used as they were in the original training materials. The team members involved in the adaptation process had experience working in mental health and could read, write, and speak the local language of study participants.

### 4.1 Elements of cultural adaptation

The purpose of using a validated cultural adaptation framework – that is, the EVM framework (42) - in this study was twofold. Firstly, to document the entire intervention adaptation processes; and secondly, to increase the cultural acceptability and effectiveness of the intervention strategies used among different target groups. We did this by making certain changes in different dimensions of the interventions that align with the culture of the study participants and the context of the health system, while retaining the core elements of the intervention that are backed by evidence informed practices (32, 42, 50, 51). While encouraging adaptations that are receptive to the requirements of study participants, it is also necessary to implement the intervention as intended. The methods dimension was adapted whereby the duration of the interventions were truncated while not making any changes to the concepts and goals. This reflects what researchers have termed the balance between fidelity and fit elements of an intervention that promotes cultural relevance of the intervention while also adhering to evidence-based practices to increase effectiveness (52, 53).

The eight dimensions that were used in the EVM framework are based on cultural adaptation facets proposed by Bernal and Rodriguez (54) in documenting the adaptation process while conceptually adhering to the intervention’s primary mechanisms of actions. Like other researchers who have pointed out that the dimensions of content and context overlap with each other (42), we also found this to be similar in the Indian setting. We found considerable commonalities across these dimension groups: language, metaphor, and content; and persons and context. While using the language dimension in the adaptation process of this study, stigmatizing labels associated to mental health conditions that emerged were quite similar to those reported from the same north Indian region (29). Under the two latter dimensions, we considered the role of gender, age, caste, and class that go into the relationships among study participants and with researchers, where social constructs such as these largely influence cultural practices (45). In one of our mixed methods formative research in the adjoining rural areas of where this present study was conducted, we found caste-based marginalization among community members and gender and age related cultural practices (45). The metaphor dimension was adapted by using a pre-existing adapted print material from an ongoing study with simple culturally relevant illustrations that were informed by formative research (45, 47). Researchers involved in cultural adaptation of psychological interventions have observed that using ethnically relevant pictorial materials identical to the study participants context improved their retention, which is referred to as surface level adaptations (55). This highlights the importance of simple, yet crucial, cultural adaptation processes (56). Engagement with participants at various stages of the study to understand their experiences and overall acceptability of these stigma reductions interventions were possible because of the cultural adaptations. However, it is critical to keep in mind that while contextually adapting complex community and health system-based interventions, some of these cultural adaptations processes are not as straightforward as they may initially appear. An important aspect of any cultural adaptation of stigma and discrimination reduction strategies should consider that the processes of contextualizing different cultural elements do not in turn reinforce or increase perceptions of stigma among study participants and service users in particular.

### 4.2 Need for cultural adaptation

The nature and extent of stigma and discrimination expressed by different groups and experienced by people with mental health conditions is greatly influenced by their cultural beliefs and practices (22–25). Despite these cultural influences, research on stigma and discrimination reduction interventions have noted that almost all interventions that are implemented in LMICs have predominantly been developed in high-income countries (HICs) based on western principles, values, and frameworks (57). This ignores how existing diverse local health systems and practices might influence the ways in which stigma and discrimination around mental health conditions are socially constructed, perceived and expressed, across culturally heterogeneous global settings (26, 27, 58). Programs in reducing stigma and discrimination of mental health conditions have made phenomenal global progress over the past few decades (1). However, unfortunately most programs are carried out in HICs (59). Most of these interventions are resource intensive and complex, and not suitable to the sociocultural contexts and health systems in LMICs making it a challenge to introduce these programs in low resource settings (32). Although it is encouraging that several interventions to address stigma and discrimination among people with mental health conditions are being implemented in LMICs (1, 60), little is known on the extent to which of these interventions have been effective, and whether these have used culturally appropriate approaches (32).

A scoping review conducted to examine the role of culture in programs to reduce stigma towards people with mental health conditions in LMICs found that only a handful of studies considered cultural elements of mental health stigma in the design and implementation of such programs (32). More importantly, the studies also lacked a systematic way of comparing cultural dimensions across and within countries. The larger Indigo Partnership study in seven sites across five LMICs (33), within which the work reported on in this study is conducted, has a dedicated work stream of culturally contextualizing interventions to reduce stigma and discrimination among people with mental health conditions. As such it will be able to provide insights into many of these research gaps. Moreover, using the EVM framework for the three interventions in the larger Indigo Partnership study provides a method to evaluate and compare cultural adaptation strategies across the seven contextually and culturally varied sites. Overall, it would be important to assess how these adaptations might possibly have an impact on the intervention outcomes, so as to identify which modifications appear to enhance intervention acceptability and effectiveness.

## 5 Conclusion

In this study stigma reduction interventions were implemented as a small-scale proof of principle interventions across different target groups in one of the North Indian sites of the Indigo collaborative research. We documented the process of culturally adapting these strategies across different dimensions and domains. Unlike previous work, we used the well validated Ecological Validity Model framework to capture these modifications and to inform the cultural adaptation and implementation science research. Stigma reduction strategies encompassing varied target groups, based on culturally appropriate adaptations and evidence, will be more applicable to stakeholders involved in the intervention in the future to achieve program acceptability and effectiveness, and possibly in taking these anti-stigma interventions to scale.

## Data availability statement

The original contributions presented in the study are included in the article/supplementary material. Further inquiries can be directed to the corresponding author.

## Ethics statement

The studies involving humans were approved by The George Institute Ethics Committee, The George Institute for Global Health (18/2020 dated 04.09.2020). Approval from the Health Ministry Screening Committee, Department of Health Research/ICMR was also sought (HMSC- 2020-10098). The studies were approved by by the KCL PNM Research Ethics Subcommittee (HR-19/20-17252). The studies were conducted in accordance with the local legislation and institutional requirements. The participants provided their written informed consent to participate in this study.

## Author contributions

MD: Writing – original draft. SK: Writing – review & editing. PCG: Writing – review & editing. SW: Writing – review & editing. BK: Writing – review & editing. GT: Writing – review & editing. PKM: Writing – review & editing.

## Footnotes

1 Mercian Daniel (MD), Senior Research Fellow, Sudha Kallakuri (SK), Research Fellow, and Santosh Chouhan Project Supervisor, The George Institute for Global Health, India.
